# The Association of Maternal Age with Birthweight and Gestational Age: A Cross-Cohort Comparison

**DOI:** 10.1111/ppe.12162

**Published:** 2014-11-18

**Authors:** María Clara Restrepo-Méndez, Debbie A Lawlor, Bernardo L Horta, Alicia Matijasevich, Iná S Santos, Ana M B Menezes, Fernando C Barros, Cesar G Victora

**Affiliations:** a Department of Epidemiology, Federal University of Pelotas; d Department of Health and Behavior, Catholic University of PelotasPelotas, Brazil; b Medical Research Council (MRC) Integrative Epidemiology Unit, University of BristolBristol, UK; c School of Social and Community Medicine, University of BristolBristol, UK

**Keywords:** pregnancy in adolescence, maternal age, low birthweight infant, preterm birth, socio-economic factors

## Abstract

**Background:**

We examined the associations of maternal age with low birthweight (LBW) and preterm birth in four cohorts from a middle- and a high-income country, where the patterning of maternal age by socio-economic position (SEP) is likely to differ.

**Methods:**

Population-based birth cohort studies were carried out in the city of Pelotas, Brazil in 1982, 1993, and 2004, and in Avon, UK in 1991 [Avon Longitudinal Study of Parents and Children (ALSPAC)]. Adjustment for multiple indicators of SEP were applied.

**Results:**

Low SEP was associated with younger age at childbearing in all cohorts, but the magnitudes of these associations were stronger in ALSPAC. Inverse associations of SEP with LBW and preterm birth were observed in all cohorts. U-shaped associations were observed between maternal age and odds of LBW in all cohorts. After adjustment for SEP, increased odds of LBW for young mothers (<20 years) attenuated to the null but remained or increased for older mothers (≥35 years). Very young (<16 years) maternal age was also associated with both outcomes even after full SEP adjustment. SEP adjusted odds ratio of having a LBW infant in women <16 years and ≥35 years, compared with 25–29 years, were 1.48 [95% confidence interval (CI) 1.00, 2.20] and 1.66 [95% CI 1.36, 2.02], respectively. The corresponding results for preterm birth were 1.80 [95% CI 1.23, 2.64)] and 1.38 [95% CI 1.15, 1.67], respectively.

**Conclusion:**

Confounding by SEP explains much of the excess risk of LBW and preterm among babies born to teenage mothers as a whole, but not for mothers aged <16 or ≥35 years. Given that the proportion of women becoming pregnant at <16 years is smaller than for those ≥35 years, the population burden is greater for older age.

Several studies have reported increased risks of low birthweight (LBW) among offspring of adolescent mothers^1^–^9^ (generally defined as women <20 years).^1^–^7^ More recently, the concern about adverse perinatal outcomes has also shifted towards older mothers as the number of births to women 35 years and older is increasing in both high-income countries (HIC) and middle-income countries (MIC).^10^–^13^ Several mechanisms have been suggested to explain these associations. With respect to adolescent mothers, it has been suggested that they are still developing and growing, and therefore, mother and offspring may compete for the supply of nutrients.^14^ At older ages, women are more likely to have pre-existing, possibly undiagnosed diseases or poor health, including reduced cardiovascular reserve, which could result in poor placentation and LBW.^11^,^12^,^15^,^16^ Furthermore, adverse perinatal outcomes in older mothers might be related to relative infertility, which could influence the likelihood of preterm births and LBW.^16^ At both ends of the age spectrum, the relationship between maternal age and adverse offspring outcomes may be strongly confounded by socio-economic position (SEP).^17^ However, this is likely to be in opposite directions, with the association between having a baby as a teenager and adverse perinatal outcomes possibly being explained by low SEP,^17^ and the generally higher SEP of older mothers concealing a biological effect of older maternal age on poor offspring outcomes.^17^

Given the difficulty of thoroughly controlling for all known confounders in observational studies, in particular regarding SEP, novel methods for exploring the association between maternal age and perinatal outcomes have been proposed.^17^,^18^ In the current paper, we have used an alternative method to explore the extent to which associations of maternal age with LBW and preterm birth might be confounded by SEP. We have focused on LBW and preterm birth because of the robust associations of both of these with infant morbidity and mortality^19^,^20^ and with lifelong adverse health outcomes,^21^ and also because these are the two commonest outcomes examined in previous studies of maternal age with adverse perinatal outcomes.^8^ We compared results across four cohorts in which we anticipate that SEP may relate differently to maternal age. The four cohorts are three birth cohorts from different time periods from Pelotas, Brazil (an MIC) and one pregnancy cohort from the South West of England (an HIC). Our assumption is that SEP will relate less strongly to maternal age at birth in the Brazilian compared with the English cohort, because young age at birth is more common in Brazil and carries less social stigma. Over the time period between the first (1982) and last (2004) of the Pelotas cohorts, Brazil has become more affluent, and with that, it adopted more Western attitudes and social behaviours. Therefore, there may be differences in the relationship of SEP to maternal age across these three Pelotas cohorts. If our assumptions are true that there are these differences in confounding structure between these four cohorts, for which some of our previous research provides evidence,^22^–^24^ then any consistency of association across the cohorts after adjusting for observed confounders is unlikely to be due to residual confounding. We are not aware of any previous studies that have used a cross-cohort comparison to explore maternal age and LBW or other perinatal outcomes.

## Methods

Pelotas is a city located in Southern Brazil where three birth cohort studies were conducted in 1982, 1993, and 2004. Avon Longitudinal Study of Parents and Children (ALSPAC) is an English birth cohort of participants born in the early 1990s. Some methodological details of the four cohorts are provided as Supplementary Information S1. More details are given elsewhere.^25^–^29^

The present analyses were restricted to singleton births, whether live or stillborn. Multiple deliveries were excluded because they are regarded as a potentially higher-risk group for LBW. Consequently, the sample size was reduced to 98.3%, 98.5%, and 98.0% from the original Pelotas cohort members, respectively, and 98.0% from the initial ALSPAC cohort participants.

Birthweight was dichotomised and those under 2500 g were classified as LBW newborns. Births before the 37th week of pregnancy were classified as preterm. Maternal age at the time of delivery was initially categorised as follows: <16, 16–19, 20–24, 25–29, 30–34 and >34 years. The number of mothers <16 years were small in all four cohorts [*n* = 65 (1.1%) in 1982; 108 (2.1%) in 1993; 114 (2.7%) in 2004, in Pelotas and 10 (0.07%), in ALSPAC], and therefore, we conducted within each cohort analyses with the two lower age groups combined into one category of <20 years. After exploring heterogeneity between the cohorts we *a priori* decided that if this was small (*P* > 0.05), we would pool results across cohorts in order to be able to specifically look at the very young (<16 years) age group, which although rare, may be at particularly high risk and is a group relatively under-researched in this area.^17^

Considering that using a single measure of SEP may not encompass the entirety of the effect of SEP on health, we decided to adjust for multiple SEP indicators to avoid as much residual confounding as possible because of unmeasured socio-economic circumstances. Three indicators of SEP – family income, maternal education, and paternal education – and maternal skin colour/ethnic origin, marital status, and parity were regarded as potential confounders. A detailed description of these covariates is provided as Supplementary Information S1.

In Pelotas, the study protocols were approved by the Medical Ethics Committee of the Federal University of Pelotas, affiliated with the Brazilian Federal Medical Council. In ALSPAC, ethical approval was obtained from the ALSPAC Law and Ethics Committee and the National Health Service Local Research Ethics Committee.

### Statistical analysis

To compare associations of SEP with maternal age and birthweight in the four cohorts, index of inequality were calculated [slope index of inequality (SII) for continuous variable (maternal age) and relative index of inequality (RII) for binary outcomes]. Through these indices, it is possible to relate outcomes with SEP indicators taking into account the different proportions in each SEP category.^30^ Each indicator of SEP is converted into a variable represented by scores from 0 (lowest SEP) to 1 (highest SEP), with each category corresponding to a score calculated as the mid-point for the proportion of participants in that category based on the cumulative distribution. The SII/RII is then obtained by regressing each outcome measure on these 0 to 1 scores.^24^ For continuous outcomes, the coefficient represents the mean difference in the outcome measure between the extremes of SEP spectra (i.e. the most deprived compared with the most affluent). For binary outcomes, the results are the odds ratio (OR) of the outcome between the extremes. Associations of maternal age with LBW were analysed using logistic regression. We present results from two models from each cohort – one in which the association of maternal age with LBW is examined without adjustment for any potential confounders and one in which we adjust for all potential confounders.

We tested between-cohort differences for the associations of SEP variables with maternal age and with LBW, and also for the main association of maternal age with LBW, using individual participant data. We combined all data from each of the four cohorts and added an indicator variable for ‘cohort’ in analyses using these pooled data. Heterogeneity in associations between the four cohorts was explored by including an interaction term for ‘cohort’. As there was no strong evidence of heterogeneity among the four cohorts, we assessed the association between maternal age and LBW based on all participants from the four cohorts combined and using ‘cohort’ as a covariate. This pooled individual participant analyses provided more precise estimates of associations, and, importantly, provided adequate statistical power for us to examine whether very young maternal age (<16) was associated with LBW and preterm birth.

In order to compare our results with most other published studies, we repeated the analyses adjusting for maternal education as the only marker of SEP in the confounder-adjusted models. This is consistent with most published studies assessing these associations.

For the main analyses presented here, we only included women in any of the four cohorts who had complete data on any variables included in the maximally adjusted multivariable model. However, to assess any potential bias caused by missing data, we also repeated the unadjusted associations using the maximal sample available and compared these results to the unadjusted associations in the main complete case analyses.

Finally, we repeated all of the above analyses with preterm birth as the outcome. In contrast to methods for measuring gestational age, which varied among the cohorts, birthweight was assessed in a uniform manner. As gestational age had a high proportion of missing values (22%) in the 1982 Pelotas cohort, hot deck multiple imputation was employed,^31^ using information from family income, maternal education, maternal skin colour, number of antenatal care visits, self-report of high blood pressure during pregnancy, and parity.

The Hosmer–Lemeshow goodness-of-fit test was applied to examine adequacy of final models of the association of maternal age with LBW and preterm birth. All analyses were performed using Stata, version 12.0 (StataCorp, College Station, Texas, USA).

## Results

The prevalence of LBW tended to be highest among adolescents in the Pelotas cohorts and among mothers older than 34 years in ALSPAC. LBW prevalence decreased in Pelotas from 1982 to 2004 in almost all maternal age groups. In all cohorts, the prevalence of preterm birth was highest among adolescents and among mothers older than 34 years. Preterm birth was less frequent in ALSPAC than in Pelotas, in almost all maternal age groups; in Pelotas, preterm birth prevalence tended to increase over time (Table 1).

**Table 1:** Prevalence of low birthweight (LBW) and preterm births in the Pelotas cohorts and ALSPAC

Cohort	Maternal age	*n*	LBW	*P*-value^a^	Preterm birth	*P*-value^a^
			% [95% CI]		% [95% CI]	
1982	<20	674	9.9 [7.7, 12.2]	<0.001	8.0 [5.7, 10.4]	0.001
	20–24	1391	6.5 [5.2, 7.8]		4.0 [2.9, 5.2]	
	25–29	1226	5.1 [3.9, 6.4]		4.5 [3.2, 5.8]	
	30–34	800	6.0 [4.3, 7.6]		4.5 [3.0, 6.1]	
	>34	485	8.0 [5.6, 10.5]		7.9 [5.2, 10.5]	
	All	4576	8.2 [7.5, 8.9]		5.5 [4.8, 6.2]	
1993	<20	802	11.6 [9.4, 13.8]	0.003	13.1 [10.8, 15.5]	0.03
	20–24	1326	8.5 [7.0, 10.0]		10.1 [8.5, 11.8]	
	25–29	1231	7.1 [5.7, 8.6]		9.2 [7.6, 10.9]	
	30–34	885	8.4 [6.5, 10.2]		10.5 [8.5, 12.6]	
	>34	517	11.4 [8.7, 14.2]		13.1 [10.2, 16.0]	
	All	4761	9.3 [8.5, 10.1]		10.8 [9.9, 11.7]	
2004	<20	584	11.1 [8.6, 13.7]	0.16	18.2 [15.0, 21.3]	0.02
	20–24	904	8.5 [6.7, 10.3]		13.6 [11.3, 15.8]	
	25–29	737	8.0 [6.0, 10.0]		12.4 [10.0, 14.7]	
	30–34	574	7.8 [5.6, 10.0]		12.2 [9.5, 14.9]	
	>34	426	10.6 [7.6, 13.5]		13.4 [10.2, 16.7]	
	All	3225	9.8 [8.9, 10.7]		13.9 [12.7, 15.0]	
ALSPAC	<20	173	5.2 [1.9, 8.5]	0.37	5.1 [1.9, 8.4]	0.36
	20–24	1121	3.6 [2.5, 4.7]		5.0 [3.8, 6.3]	
	25–29	3081	3.1 [2.5, 3.7]		3.8 [3.1, 4.4]	
	30–34	2442	3.2 [2.5, 3.8]		3.9 [3.1, 4.6]	
	>34	863	4.1 [2.7, 5.4]		4.1 [2.8, 5.4]	
	All	7680	3.3 [2.9, 3.7]		4.0 [3.6, 4.5]	

a Chi-squared for heterogeneity.

Table S1 shows the SII for maternal age, expressing the differences between the extremes of the income and parental education spectra and therefore allowing a direct comparison of the magnitudes of the associations in the four cohorts. Mean maternal age tended to be directly associated with SEP in the four cohorts. The only exception to this pattern was the inverse association of paternal education with maternal age in the Pelotas 1982 cohort. Although the direction of these associations was similar for all four cohorts, the magnitude of associations tended to be stronger in ALSPAC than any of the three Pelotas cohorts, especially for maternal and paternal education (*P* < 0.001 for interaction between SEP variables and study). In contrast, the associations of SEP indicators with LBW (Table S2) had similar magnitude across all four cohorts (*P*-value for interaction between both income and maternal education and study = 0.2; and *P* = 0.8 for interaction between paternal education and study = 0.8).

Table 2 displays the ORs and 95% confidence interval of the associations between maternal age and LBW for each individual study. Adjustment for confounders tended to attenuate the ORs associated with all age groups except that of mothers 30 years or older, for which adjustment increased the OR. The reduction in the risk for adolescent mothers following confounder adjustment was largest in the Pelotas 2004 and ALSPAC studies. The increase in risk for older mothers following adjustment was particularly noticeable in ALSPAC.

**Table 2:** Associations of maternal age with low birthweight (LBW) in the Pelotas cohorts and ALSPAC

Cohort	Maternal age	Unadjusted OR [95% CI]	Adjusted OR^a^ [95% CI]	Adjusted OR^b^ [95% CI]
		*P* = 0.002	*P* = 0.14	*P* = 0.08
1982	<20	2.04 [1.43, 2.92]	1.42 [0.95, 2.13]	1.63 [1.10, 2.41]
	20–24	1.30 [0.93, 1.80]	1.15 [0.82, 1.61]	1.20 [0.86, 1.68]
	25–29	1.00	1.00	1.00
	30–34	1.18 [0.80, 1.74]	1.21 [0.82, 1.79]	1.19 [0.81, 1.76]
	>34	1.62 [1.07, 2.45]	1.65 [1.08, 2.52]	1.57 [1.03, 2.39]
		*P* = 0.003	*P* = 0.02	*P* = 0.03
1993	<20	1.70 [1.25, 2.31]	1.30 [0.93, 1.84]	1.36 [0.97, 1.91]
	20–24	1.21 [0.91, 1.62]	1.11 [0.82, 1.49]	1.12 [0.83, 1.50]
	25–29	1.00	1.00	1.00
	30–34	1.19 [0.86, 1.64]	1.31 [0.95, 1.81]	1.27 [0.92, 1.75]
	>34	1.67 [1.18, 2.37]	1.77 [1.25, 2.52]	1.73 [1.22, 2.46]
		*P* = 0.17	*P* = 0.29	*P* = 0.40
2004	<20	1.44 [0.99, 2.08]	0.94 [0.61, 1.44]	1.01 [0.67, 1.53]
	20–24	1.07 [0.75, 1.52]	0.91 [0.64, 1.31]	0.97 [0.68, 1.39]
	25–29	1.00	1.00	1.00
	30–34	0.98 [0.65, 1.46]	1.00 [0.66, 1.51]	0.99 [0.66, 1.49]
	>34	1.36 [0.90, 2.04]	1.45 [0.95, 2.20]	1.43 [0.94, 2.17]
		*P* = 0.28	*P* = 0.03	*P* = 0.22
ALSPAC	<20	1.93 [1.02, 3.66]	0.82 [0.39, 1.75]	0.98 [0.46, 2.08]
	20–24	1.17 [0.82, 1.68]	0.83 [0.56, 1.23]	0.96 [0.66, 1.42]
	25–29	1.00	1.00	1.00
	30–34	1.01 [0.75, 1.36]	1.29 [0.94, 1.76]	1.20 [0.88, 1.64]
	>34	1.28 [0.87, 1.89]	1.75 [1.16, 2.65]	1.61 [1.07, 2.43]

a Adjusted for confounding factors: family income, maternal education, paternal education, skin colour/ethnic group, parity, and living with a partner.

b Adjusted for maternal education, skin colour/ethnic group, parity, and living with a partner.

The attenuation in the odds of preterm birth in adolescent mothers following confounder adjustment was also largest in the Pelotas 2004 and ALSPAC studies. The increase in odds for older mothers following adjustment was particularly noticeable in the 1982 Pelotas cohort. Further, older mothers had consistently higher odds for preterm birth in comparison with women aged 25–29 years, but the magnitude of the difference decreased from the 1982 to the 2004 cohort (Table 3).

**Table 3:** Associations of maternal age with preterm births in the Pelotas cohorts and ALSPAC

Cohort	Maternal age	Unadjusted OR [95% CI]	Adjusted OR^a^ [95% CI]	Adjusted OR^b^ [95% CI]
		*P* = 0.001	*P* = 0.006	*P* = 0.003
1982	<20	1.75 [1.21, 2.53]	1.64 [1.00, 2.69]	1.70 [1.05, 2.77]
	20–24	0.90 [0.63, 1.28]	0.85 [0.55, 1.31]	0.85 [0.55, 1.30]
	25–29	1.00	1.00	1.00
	30–34	1.03 [0.69, 1.53]	1.01 [0.63, 1.63]	1.03 [0.64, 1.66]
	>34	1.66 [1.09, 2.52]	1.81 [1.11, 2.93]	1.87 [1.16, 3.01]
		*P* = 0.03	*P* = 0.08	*P* = 0.07
1993	<20	1.43 [1.10, 1.86]	1.32 [0.96, 1.81]	1.35 [0.99, 1.85]
	20–24	1.09 [0.85, 1.40]	1.05 [0.80, 1.38]	1.06 [0.81, 1.39]
	25–29	1.00	1.00	1.00
	30–34	1.14 [0.86, 1.50]	1.19 [0.89, 1.60]	1.19 [0.89, 1.60]
	>34	1.46 [1.08, 1.98]	1.49 [1.08, 2.07]	1.49 [1.08, 2.07]
		*P* = 0.006	*P* = 0.70	*P* = 0.56
2004	<20	1.56 [1.20, 2.03]	1.24 [0.87, 1.76]	1.30 [0.92, 1.84]
	20–24	1.15 [0.89, 1.48]	1.02 [0.76, 1.38]	1.06 [0.79, 1.42]
	25–29	1.00	1.00	1.00
	30–34	1.00 [0.75, 1.34]	0.98 [0.70, 1.37]	0.98 [0.70, 1.38]
	>34	1.06 [0.78, 1.45]	1.10 [0.76, 1.58]	1.11 [0.78, 1.60]
		*P* = 0.007	*P* = 0.74	*P* = 0.22
ALSPAC	<20	1.37 [0.98, 1.92]	1.01 [0.48, 2.13]	1.10 [0.52, 2.30]
	20–24	1.30 [1.06, 1.59]	1.17 [0.83, 1.64]	1.26 [0.90, 1.75]
	25–29	1.00	1.00	1.00
	30–34	0.89 [0.73, 1.09]	1.14 [0.86, 1.52]	1.12 [0.84, 1.48]
	>34	1.09 [0.84, 1.43]	1.26 [0.85, 1.88]	1.22 [0.83, 1.81]

a Adjusted for confounding factors: family income, maternal education, paternal education, skin colour/ethnic group, parity, and living with a partner.

b Adjusted for maternal education, skin colour/ethnic group, parity, and living with a partner.

**Figure 1:**
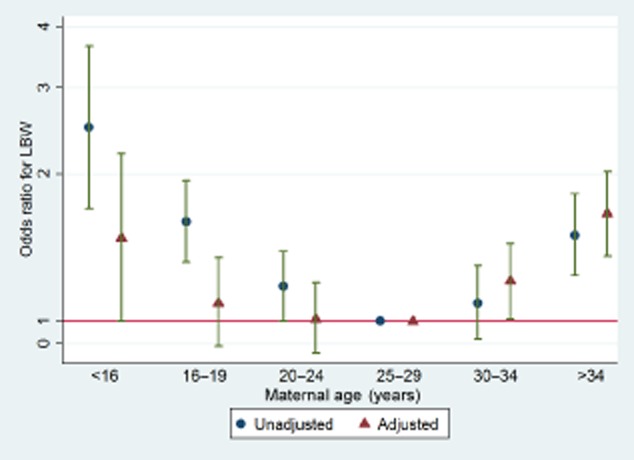
Combined unadjusted and adjusted odds ratio (OR) of low birthweight (LBW) by maternal age (including the three Pelotas cohorts and ALSPAC). *P*-values for interaction between associations of age and study with LBW were 0.8 for the unadjusted analyses and 0.9 for the adjusted analyses (i.e. suggesting that associations of age with LBW were similar in all four studies).

**Figure 2:**
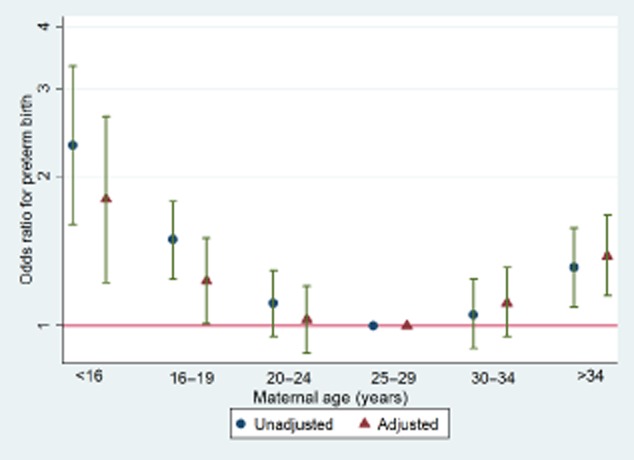
Combined unadjusted and adjusted odds ratio (OR) of preterm birth by maternal age (including the three Pelotas cohorts and ALSPAC). Unadjusted and adjusted *P*-values for interaction between maternal age and study were 0.5 and 0.7, respectively.

There were too few adolescent mothers aged <16 years in each individual cohort to reliably estimate the odds of outcomes compared with the reference group. Since there was no strong evidence for the interaction between maternal age categories and LBW by cohort study (unadjusted and adjusted model had *P* = 0.8 and *P* = 0.9, respectively), we combined the cohorts. Figure 1 shows that with all four studies combined, there are important increases in the odds of LBW in those <16 years and >34 years, compared with the reference group of 25–29 years (see also Table S3). In addition, adjustment for confounders tended to attenuate the ORs associated with all age groups except that of mothers 30 years or older, for which adjustment increased the OR. In a similar manner, associations of maternal age with preterm birth did not differ between the four studies. Results with preterm birth as the outcome were generally similar to those for LBW (Figure 2). However after adjustment for confounders, both adolescent and older mothers continued to show a strong evidence of increased odds for preterm birth (see also Table S3).

In comparison with adjusting for all potential SEP indicators in our studies, when we adjusted only for confounding by maternal education, the association of young maternal age with LBW attenuated less and that of older maternal age with LBW increased less in the Pelotas 1982 cohort, but for all of the other cohorts, there was no marked difference between the two models (Table 2). For preterm birth, adjusting only for maternal education produced broadly similar findings to adjusting for all SEP confounders in all four cohorts.

Analyses with maximum sample showed similar magnitudes of association between LBW and preterm birth with maternal age for all four cohorts (Table S4). In addition, when comparing results with and without imputation data for gestational age for the 1982 Pelotas cohort, we observed similar magnitudes of association between preterm birth and maternal age.

## Comment

We found slightly different associations of maternal age with odds of LBW and preterm birth in three cohorts from different time periods (early 80s, 90s, and 2000s) based in Brazil and one from the early 90s based in England. In the 1982 and 1993 Pelotas cohorts, maternal age younger than 20 years and older than 34 years, compared with the reference group of 25–29 years, had increased odds of LBW. After adjustment for SEP, the increased odds of LBW for younger mothers (<20 years) attenuated to the null, and the higher odds for older mothers increased for the 1993 Pelotas cohort as well as for ALSPAC. This confirms our hypothesis that the association of young maternal age with LBW was positively and strongly confounded by SEP, whereas for older maternal age, SEP acts as a negative (masking) confounder that attenuates the association in unadjusted analyses. In a similar manner, the higher odds of preterm birth for older mothers increased after adjustment for SEP in the three Pelotas cohorts.

The comparison of confounding structures in the four cohorts shows that SEP was positively associated with maternal age in all cohorts, but the association was particularly strong in ALSPAC. Therefore, one would expect more marked changes from the unadjusted to the adjusted ORs in ALSPAC than in Pelotas, and this was indeed the case. For instance, in ALSPAC, the unadjusted OR of 1.93 for adolescent mothers was reduced to 0.82, whereas for older mothers, the corresponding values were 1.28 and 1.75. Changes from the unadjusted to the adjusted ORs in Pelotas were considerably less marked.

Changes in the socio-economic characteristics of the mothers as well as the reproductive and health care conditions over time in Pelotas may explain the differences in the association of maternal age with LBW and preterm births among the Pelotas cohorts. Improvements in maternal education, household conditions, and presence of consumer goods as well as increased coverage of antenatal care from 1982 to 2004 may explain the lower risk for LBW in the 2004 Pelotas cohort.^32^ For instance, adolescents had lower family income, lower parity, and were less likely to live with a partner, compared with mothers aged 20–29 years, in the three Pelotas cohorts. In 1982 and 1993, adolescents also had lower schooling, but by 2004, this trend had reversed.^33^ This improvement in the education of adolescents may explain why the associations of younger maternal age with LBW and preterm birth were less marked in the 2004 cohort than in the 1982 and 1993 cohorts. Furthermore, smaller birth interval and higher parity^13^ may explain the greater risk for LBW among older women in the 1982 and 1993 Pelotas cohort. In contrast, the excessive medicalisation of pregnancy and delivery^32^ – especially among more affluent groups of SEP – may be responsible for the greater prevalence for preterm birth in the 2004 Pelotas cohort and for the absent of association of older maternal age with LBW and preterm births.^32^,^34^ Because of the improvements of socio-economic characteristics of the mothers in Brazil over time, one would expect that findings from the 2004 Pelotas cohort would be more similar to those from ALSPAC, but this was not the case. Again, the increase of excessive medicalisation over time in Brazil may explain the different pattern observed in the 2004 Pelotas cohort.

To test how effective controlling for maternal education, instead of the full set of SEP measures, was in reducing residual confounding, we ran analyses for the four cohorts and compared both sets of estimates. The magnitudes of odds for LBW and preterm birth were higher in the model adjusted for maternal education relative to the full model that also included income and paternal education for all cohorts. This suggests that, at least in some previous studies, controlling for maternal education alone may not have been sufficient to eliminate socio-economic confounding and may explain why some of the reports in the literature are in disagreement with our present findings.^17^,^35^

An additional important finding of our study is that by combining all four cohorts, we had adequate statistical power to examine whether very young maternal age (<16 years) was associated with adverse perinatal outcomes. Very few studies have sufficient statistical power to precisely estimate the association between very young maternal age at birth and adverse perinatal outcomes. Our results show that although in general much of the association of pregnancy aged 20 years or younger with LBW and preterm birth is spurious and explained by SEP, confounding this finding masks an important increased risk of adverse outcomes in women aged 16 years or younger even after adjustment for SEP. The suggestion that adverse perinatal outcomes occur in women of young age because they are still growing and are hence unable to obtain sufficient nutrients for both their own growth and development as well as that of the fetus is likely to be more the case for very young mothers (<16 years) than those who are 16–19 years. These findings are consistent with the small number of other studies that have been able to examine associations with very young maternal age (defined as either <16 or <14 years).^1^,^5^,^36^

The strengths of these analyses include the population-based samples from four prospective studies that allowed a comparison between cohorts from HIC and MIC, and the detailed assessment of maternal characteristics, which enabled us to control for three indicators of SEP, as well as other factors (maternal skin colour, parity) that might confound the associations of maternal age with LBW and preterm birth.

Mothers who were not included in the main analyses (restricted sample) were more likely to be from the younger age groups (<20 and 20–24 years) in ALSPAC; therefore, we cannot rule out the possibility that our findings can be biased towards the null among younger mothers. However, results were similar for the unadjusted analyses when we used the maximum sample compared with the restricted sample for all four cohorts.

Our findings point to different mechanisms, as well as different social patterning, being responsible for the associations of young maternal age with LBW compared with those of older maternal age. They suggest that very young maternal age is associated with higher risk of LBW and preterm birth, but that the apparent increase in risk for mothers aged 16–19 years is explained by their socio-economic, rather than biological conditions. For this group, a focus on socio-economic inequalities rather than younger maternal age *per se* might yield greater population benefit, whereas for older maternal age, our findings highlight the need for further research to explore biological mechanisms underlying these associations. Although there is considerable research and media coverage regarding the potential detrimental consequences of adolescent pregnancy, the possible adverse impact of delaying a first birth until older ages is much less discussed in the media or health journals. Given our results, it might be time for public discussion to consider adverse outcomes of older maternal age at birth in more detail.

## Supporting Information

Additional Supporting Information may be found in the online version of this article at the publisher's web-site: **Table S1.** Associations of indicators of socio-economic position (SEP) with maternal age in the Pelotas cohorts and ALSPAC. **Table S2.** Associations of indicators of socio-economic position (SEP) with low birthweight (LBW) and preterm birth in the Pelotas cohorts and ALSPAC. **Table S3.** Combined unadjusted and adjusted odds ratio (OR) and 95% CI of low birthweight (LBW) and preterm birth by maternal age (including the three Pelotas cohorts and ALSPAC). **Table S4.** Associations of maternal age with low birthweight (LBW) and preterm birth in the Pelotas and ALSPAC cohorts (maximum sample). **Supplementary Information S1.** Description of covariates regarded as potential confounders in the association of maternal age with low birthweight and preterm birth for Pelotas cohorts and ALSPAC.
