# Supplementary material for: The Association of Maternal Age with Birthweight and Gestational Age: A Cross-Cohort Comparison
Source: Paediatr Perinat Epidemiol. 2014 Nov 18;29(1):31–40. doi: 10.1111/ppe.12162 (PMC4296235; doi:10.1111/ppe.12162)
Supplement: Supplementary file 1 [file ppe0029-0031-sd1.docx]

**Supplementary Web Information 1**. Description of covariables regarded as potential confounders in the association of maternal age with low birthweight and preterm birth for Pelotas cohorts and ALSPAC.

**Web-table 1.** Associations of indicators of socio-economic position (SEP) with maternal age in the Pelotas cohorts and ALSPAC.

**Web-table 2.** Associations of indicators of socio-economic position (SEP) with low birthweight (LBW) and preterm birth in the Pelotas cohorts and ALSPAC.

**Web-table 3.** Combined unadjusted and adjusted odds ratio (OR) and 95% CI of LBW and preterm birth by maternal age (including the three Pelotas cohorts and ALSPAC).

**Web-table 4.** Associations of maternal age with LBW and preterm birth in the Pelotas and ALSPAC cohorts (maximum sample).

**Supplementary Web Information 1**

**The association of maternal age with birthweight and gestational age: a cross-cohort comparison**

Restrepo-Méndez MC, Lawlor DA, Horta BL, Matijasevich A, Santos IS, Menezes AMB, Barros FC, Victora CG

**METHODS**

**The Pelotas Birth Cohorts**

All births to mothers residing in the urban area of the city were eligible and 6011, 5304 and 4287 newborns, respectively, were enrolled to each study. Daily visits to all maternity wards were carried out in order to identify births that took place in hospitals (>98% of all deliveries) and mothers were interviewed with standardized questionnaires. Data collection was conducted in all three cohorts using similar methodology. Newborns were weighed soon after birth by maternity staff using pediatric scales regularly calibrated by members of the research team. Gestational age was calculated based on the date of last menstrual period, and children whose birthweight was outside the range of acceptable weights for that age were considered to be of unknown gestational age. In 1993 and 2004 Pelotas cohorts, an algorithm proposed by the National Center for Health Statistics (NCHS) was used, employing an estimated age based on the last menstrual period (LMP). In case the last menstrual period-based gestational age was unknown or inconsistent, the clinical maturity estimate based on the Dubowitz method was adopted.

***Socioeconomic variables****:* For the three Pelotas cohorts, family income in the month prior to delivery was collected at the perinatal interview and expressed in five categories (≤1.0; 1.1-3.0; 3.1-6.0; 6.1-10.0 and >10.0) of minimum wage per month (minimum wage per month is a standardized measure of income, one minimum wage was worth about US $50 in 1982, US$60 in 1993, and US$80 in 2004). Maternal and paternal education were recorded as the number of completed years of education, with these data collected from parents at the time of delivery and categorised in 0 to 4, 5 to 8 and ≥ 9 completed years of formal education. The former category represents mothers who completed compulsory education and attained additional years of formal education (28% in 1982, 26% in 1993 and 47% in 2004).

***Additional potential confounders:*** Mother’s skin colour was self-reported. Because women in the black and “other” skin colour groups had similar socio-demographic characteristics in all cohorts, we collapsed women into one of two groups (white or black/mixed ethnic origin).

**The Avon Longitudinal Study of Parents and Children (ALSPAC)**

Pregnant women living in three health districts in the former county of Avon (centred around the city of Bristol in the South West of England) with an expected date of delivery between 1st April 1991 and 31st December 1992 were eligible to enroll and a total of 14,541 pregnancies to 13,761 women were recruited. Pregnancy and perinatal data was obtained from questionnaires at various points during pregnancy and after delivery, as well as from clinical records. Birthweights were abstracted from hospital records; these will have been entered by midwife staff who weight infants on the labour ward immediately after birth. Gestational age was recorded using LMP, paediatric assessment, obstetric assessment and ultrasound assessment. In general, the LMP was used if the mother was certain of it and there were no other clinical suggestions that this was erroneous. If the LMP date was considered unreliable, then the earliest ultrasound measurement was most likely to be taken.

***Socioeconomic variables****:* Participants were asked to indicate from one of five categories of income per week they belonged to (<£100; £100-199; £200-299; £300-399; >400) in a questionnaire completed at 33 months after delivery. Information on education was assessed at 32 weeks of gestation and categorised in three groups according to increasing levels of achievement: none / CSE only (Certificate of Secondary Education a lower level national exam usually taken at 16 years which is the minimum school leaving age) / vocational, O-level (Ordinary-level higher level national exams usually taken at 16 years), and A-level (Advanced-level national school exams usually taken at 18 years and required for university entry) / and university degree. The former category represents 44% of the sample.

***Additional potential confounders:*** Mother’s skin colour or ethnic origin was self-reported. Because of small numbers of women who were from ethnic groups other than white European in ALSPAC, we collapsed women into one of two groups (white or black/mixed ethnic origin).

In all four cohorts, Pelotas and ALSPAC, marital status was classified into mothers living with or without a partner in all cohorts. Parity was defined as the number of previous births, including stillbirths.

| **Web-Table 1. Associations of indicators of socio-economic position with maternal age in the Pelotas cohorts and ALSPAC** | | | | | | | | | | | | | | | | | | | | | | | | |  |  |  |  |  |
| --- | --- | --- | --- | --- | --- | --- | --- | --- | --- | --- | --- | --- | --- | --- | --- | --- | --- | --- | --- | --- | --- | --- | --- | --- | --- | --- | --- | --- | --- |
| **Outcome** | **Indicator of socio-economic position** | **1982 Pelotas** | | | | | | | | | **1993 Pelotas** | | | | | | **2004 Pelotas** | | | | | | **ALSPAC** | | | | | | **P-value for interaction between study and SEP indicator** |
|  |  | **N** | **SII** | | **95% CI** | | | | | **P value** | **N** | **SII** | **95% CI** | | | **P value** | **N** | **SII** | **95% CI** | | | **P value** | **N** | **SII** | **95% CI** | | | **P value** |  |
| Maternal | Income | 4576 | 4.99 | | 4.33 | | , | 5.65 | | <0.001 | 4775 | 4.09 | 3.44 | , | 4.74 | <0.001 | 3229 | 5.93 | 5.09 | , | 6.77 | <0.001 | 7763 | 5.43 | 5.08 | , | 5.78 | <0.001 | <0.001 |
| age, y | Maternal education | 4576 | 0.66 | | 0.00 | | , | 1.32 | | 0.05 | 4775 | 2.76 | 2.09 | , | 3.43 | <0.001 | 3229 | 1.57 | 0.69 | , | 2.44 | <0.001 | 7763 | 4.47 | 4.09 | , | 4.84 | <0.001 | <0.001 |
|  | Paternal education | 4576 | -0.72 | | -1.38 | | , | -0.05 | | 0.03 | 4775 | 0.91 | 0.23 | , | 1.59 | 0.009 | 3229 | 0.95 | 0.07 | , | 1.83 | 0.03 | 7763 | 4.20 | 3.82 | , | 4.57 | <0.001 | <0.001 |
|  |  |  |  | |  | |  |  | |  |  |  |  |  |  |  |  |  |  |  |  |  |  |  |  |  |  |  |  |
| For maternal age as an outcome (continuous variable), the slope index of inequality (SII) was assessed using linear regression (2-side P value); associations reflect mean difference in maternal age in the highest socioeconomic position (SEP) level vs the lowest. | | | | | | | | | | | | | | | | | | | | | | | | | | | | | |
|  | |  | |  | |  |  |  |  | |  |  |  |  |  |  |  |  |  |  |  |  |  |  |  |  |  |  |  |

| **Web-table 2. Associations of indicators of socio-economic position with LBW and preterm births in the Pelotas cohorts and ALSPAC** | | | | | | | | | | | | | | | | | | | | | | | | | | |
| --- | --- | --- | --- | --- | --- | --- | --- | --- | --- | --- | --- | --- | --- | --- | --- | --- | --- | --- | --- | --- | --- | --- | --- | --- | --- | --- |
|  |  | **1982 Pelotas** | | | | | | **1993 Pelotas** | | | | | | **2004 Pelotas** | | |  |  |  | **ALSPAC** | | | | | | **P-value for interaction between study and SEP indicator** |
| **Child outcome** | **Indicator of socio-economic position** | **N** | **RII** | **95% CI** | | | **P value** | **N** | **RII** | **95% CI** | | | **P value** | **N** | **RII** | **95% CI** | | | **P value** | **N** | **RII** | **95% CI** | | | **P value** |  |
| Low | Income | 4576 | 0.28 | 0.18 | , | 0.44 | <0.001 | 4761 | 0.53 | 0.37 | , | 0.77 | 0.001 | 3225 | 0.41 | 0.26 | , | 0.64 | <0.001 | 7680 | 0.35 | 0.22 | , | 0.56 | <0.001 | 0.2 |
| birthweight | Maternal education | 4576 | 0.51 | 0.33 | , | 0.79 | 0.003 | 4761 | 0.37 | 0.25 | , | 0.53 | <0.001 | 3225 | 0.49 | 0.31 | , | 0.77 | 0.002 | 7680 | 0.71 | 0.45 | , | 1.14 | 0.16 | 0.2 |
|  | Paternal education | 4576 | 0.53 | 0.34 | , | 0.82 | 0.004 | 4761 | 0.45 | 0.31 | , | 0.66 | <0.001 | 3225 | 0.41 | 0.26 | , | 0.64 | <0.001 | 7680 | 0.53 | 0.33 | , | 0.85 | 0.008 | 0.8 |
|  |  |  |  |  |  |  |  |  |  |  |  |  |  |  |  |  |  |  |  |  |  |  |  |  |  |  |
| Preterm | Income | 4548 | 0.85 | 0.53 | , | 1.37 | 0.51 | 4705 | 0.63 | 0.45 | , | 0.88 | 0.007 | 3179 | 0.53 | 0.36 | , | 0.77 | 0.001 | 7763 | 0.62 | 0.41 | , | 0.93 | 0.02 | 0.7 |
| birth | Maternal education | 4548 | 1.05 | 0.65 | , | 1.69 | 0.83 | 4705 | 0.54 | 0.38 | , | 0.77 | 0.001 | 3179 | 0.51 | 0.35 | , | 0.75 | 0.001 | 7763 | 0.98 | 0.64 | , | 1.50 | 0.92 | 0.06 |
|  | Paternal education | 4548 | 0.74 | 0.46 | , | 1.2 | 0.22 | 4705 | 0.57 | 0.4 | , | 0.81 | 0.002 | 3179 | 0.39 | 0.26 | , | 0.57 | <0.001 | 7763 | 0.76 | 0.5 | , | 1.17 | 0.22 | 0.09 |
|  |  |  |  |  |  |  |  |  |  |  |  |  |  |  |  |  |  |  |  |  |  |  |  |  |  |  |
| For low birht weight and preterm birth (binary outcomes), the relative index of inequality was assessed using logistic regression (2-sided P values); associations reflect odds ratios for outcomes in the highest | | | | | | | | | | | | | | | | | | | | | | | | | | |
| socioeconomic position (SEP) level vs the lowest level. | | | | | | |  |  |  |  |  |  |  |  |  |  |  |  |  |  |  |  |  |  |  |  |

| **Web-table 3. Combined unadjusted and adjusted odds ratio (OR) and 95% CI of LBW and preterm birth by maternal age (including the three Pelotas cohorts and ALSPAC).** | | | | | | | | | | | | | |
| --- | --- | --- | --- | --- | --- | --- | --- | --- | --- | --- | --- | --- | --- |
| **Outcome** | **Maternal age** | **Combined**^*^  **unadjusted OR** | **95% CI** | | | **Combined**^*^  **adjusted OR¹** | **95% CI** | | | **Combined**^*^  **adjusted OR²** | **95% CI** | | |
|  |  |  | P<0.001 | | |  | P<0.001 | | |  | P<0.001 | | |
| LBW | <16 | 2.49 | 1.70 | , | 3.65 | 1.48 | 1.00 | , | 2.20 | 2.48 | 1.68 | , | 3.68 |
|  | 16-19 | 1.60 | 1.32 | , | 1.94 | 1.09 | 0.89 | , | 1.35 | 1.65 | 1.36 | , | 2.02 |
|  | 20-24 | 1.18 | 1.00 | , | 1.39 | 1.01 | 0.86 | , | 1.20 | 1.28 | 1.09 | , | 1.51 |
|  | 25-29 | 1.00 |  |  |  | 1.00 |  |  |  | 1.00 |  |  |  |
|  | 30-34 | 1.09 | 0.92 | , | 1.3 | 1.21 | 1.01 | , | 1.44 | 1.14 | 0.96 | , | 1.35 |
|  | >34 | 1.50 | 1.24 | , | 1.82 | 1.66 | 1.36 | , | 2.02 | 1.71 | 1.68 | , | 2.07 |
|  |  |  |  |  |  |  |  |  |  |  |  |  |  |
|  |  |  | P<0.001 | | |  | P<0.001 | | |  | P<0.001 | | |
| Preterm | <16 | 2.31 | 1.60 | , | 3.33 | 1.80 | 1.23 | , | 2.64 | 1.86 | 1.27 | , | 2.73 |
| births | 16-19 | 1.49 | 1.24 | , | 1.79 | 1.23 | 1.01 | , | 1.50 | 1.28 | 1.05 | , | 1.56 |
|  | 20-24 | 1.11 | 0.95 | , | 1.30 | 1.03 | 0.88 | , | 1.20 | 1.05 | 0.90 | , | 1.23 |
|  | 25-29 | 1.00 |  |  |  | 1.00 |  |  |  | 1.00 |  |  |  |
|  | 30-34 | 1.05 | 0.90 | , | 1.24 | 1.11 | 0.95 | , | 1.31 | 1.10 | 0.93 | , | 1.29 |
|  | >34 | 1.31 | 1.09 | , | 1.58 | 1.38 | 1.15 | , | 1.67 | 1.37 | 1.13 | , | 1.65 |
|  |  |  |  |  |  |  |  |  |  |  |  |  |  |
| ¹Adjusted for confounding factors: family income, maternal education, paternal education, skin color/ethnic group, parity, and living with a partner. | | | | | | | | | | | | | |
| ² Adjusted for maternal education, skin color/ethnic group, parity and living with a partner. | | | | | | | | | | | | | |
| ^*^Combined cohort studies: Unadjusted and adjusted P-values for interaction between maternal age and cohort study were 0.8 and 0.9 for LBW and 0.5 and 0.7 for preterm birth, respectively | | | | | | | | | | | | | |
| 2-sided P values | | | | | | | | | | | | | |

| **Web-table 4. Associations of maternal age with low birthweight (LBW) and preterm birth** | | | | | | | | | | | | |
| --- | --- | --- | --- | --- | --- | --- | --- | --- | --- | --- | --- | --- |
| **in the Pelotas cohorts and ALSPAC (maximum sample)** | | | | | | | | | | | | |
|  |  |  | **LBW** | | | | |  | **Preterm birth** | | | |
| **Cohort** | **Maternal age** | **N^*^** | **Unadjusted OR** | **95% CI** | | | | **N^**^** | **Unadjusted OR** | **95% CI** | | |
|  |  |  |  | P<0.001 | | | |  |  | P<0.001 | | |
| 1982 | <20 | 908 | 2.17 | 1.63 | | , | 2.89 | 893 | 1.65 | 1.20 | , | 2.29 |
|  | 20-24 | 1819 | 1.41 | 1.08 | | , | 1.84 | 1796 | 0.93 | 0.68 | , | 1.26 |
|  | 25-29 | 1559 | 1.00 |  | |  |  | 1540 | 1.00 |  |  |  |
|  | 30-34 | 957 | 1.21 | 0.88 | | , | 1.67 | 946 | 1.06 | 0.74 | , | 1.51 |
|  | >34 | 567 | 1.61 | 1.13 | | , | 2.28 | 566 | 1.61 | 1.11 | , | 2.34 |
|  |  |  |  |  | |  |  |  |  |  |  |  |
|  |  |  |  | P=0.011 | | | |  |  | P=0.03 | | |
| 1993 | <20 | 913 | 1.54 | 1.15 | | , | 2.05 | 900 | 1.43 | 1.10 | , | 1.86 |
|  | 20-24 | 1436 | 1.20 | 0.91 | | , | 1.57 | 1411 | 1.09 | 0.85 | , | 1.40 |
|  | 25-29 | 1324 | 1.00 |  | |  |  | 1315 | 1.00 |  |  |  |
|  | 30-34 | 936 | 1.18 | 0.87 | | , | 1.60 | 926 | 1.14 | 0.86 | , | 1.50 |
|  | >34 | 567 | 1.62 | 1.17 | | , | 2.25 | 561 | 1.46 | 1.08 | , | 1.98 |
|  |  |  |  |  | |  |  |  |  |  |  |  |
|  |  |  |  | P=0.411 | | | |  |  | P=0.006 | | |
| 2004 | <20 | 800 | 1.32 | 0.96 | | , | 1.81 | 794 | 1.56 | 1.20 | , | 2.03 |
|  | 20-24 | 1141 | 1.19 | 0.89 | | , | 1.61 | 1135 | 1.15 | 0.89 | , | 1.48 |
|  | 25-29 | 943 | 1.00 |  | |  |  | 942 | 1.00 |  |  |  |
|  | 30-34 | 744 | 1.05 | 0.75 | | , | 1.48 | 742 | 1.00 | 0.75 | , | 1.34 |
|  | >34 | 564 | 1.27 | 0.89 | | , | 1.80 | 562 | 1.06 | 0.78 | , | 1.45 |
|  |  |  |  |  | |  |  |  |  |  |  |  |
|  |  |  |  | P<0.001 | | | |  |  | P=0.007 | | |
| ALSPAC | <20 | 641 | 2.17 | 1.59 | | , | 2.95 | 654 | 1.37 | 0.98 | , | 1.92 |
|  | 20-24 | 2621 | 1.45 | 1.18 | | , | 1.80 | 2659 | 1.30 | 1.06 | , | 1.59 |
|  | 25-29 | 5232 | 1.00 |  | |  |  | 5297 | 1.00 |  |  |  |
|  | 30-34 | 3691 | 0.88 | 0.70 | | , | 1.09 | 3736 | 0.89 | 0.73 | , | 1.09 |
|  | >34 | 1343 | 1.31 | 1.00 | | , | 1.72 | 1364 | 1.09 | 0.84 | , | 1.43 |
|  |  |  |  |  | |  |  |  |  |  |  |  |
|  |  |  |  | P<0.001 | | | |  |  | P<0.001 | | |
| Combined | <16 | 295 | 2.96 | 2.12 | | , | 4.15 | 270 | 3.41 | 2.48 | , | 4.67 |
|  | 16-19 | 2967 | 2.06 | 1.77 | | , | 2.38 | 2730 | 1.89 | 1.64 | , | 2.19 |
|  | 20-24 | 7017 | 1.48 | 1.30 | | , | 1.68 | 6638 | 1.29 | 1.14 | , | 1.46 |
|  | 25-29 | 9058 | 1.00 |  | |  |  | 8828 | 1.00 |  |  |  |
|  | 30-34 | 6328 | 1.03 | 0.89 | | , | 1.18 | 6188 | 1.00 | 0.88 | , | 1.14 |
|  | >34 | 3041 | 1.57 | 1.34 | | , | 1.84 | 2939 | 1.41 | 1.21 | , | 1.65 |
|  |  |  |  |  | |  |  |  |  |  |  |  |
| ^*^ Number of mothers with information for birthweight | | | | | | | |  |  |  |  |  |
| ^**^ Number of mothers with information for gestational age | | | | | | | | |  |  |  |  |
| 2-sided P values | |  |  | |  |  |  |  |  |  |  |  |
